# Unmasking POEMS (Polyneuropathy, Organomegaly, Endocrinopathy, Monoclonal Gammopathy, and Skin Changes) Syndrome in a Heart Failure Patient: A Diagnostic Challenge

**DOI:** 10.7759/cureus.81075

**Published:** 2025-03-24

**Authors:** Frances Ogunnaya, Mohamed E Kambal, Thowaiba E Ali, Dina Elsayed, Jose Bustillo

**Affiliations:** 1 Internal Medicine, Newark Beth Israel Medical Center, Newark, USA; 2 Internal Medicine, University of Khartoum, Khartoum, SDN; 3 Healthcare Administration, University of Tennessee at Chattanooga, Chattanooga, USA; 4 Medicine-Pediatrics, Newark Beth Israel Medical Center, Newark, USA

**Keywords:** castleman disease, heart failure, monoclonal gammopathies, peripheral neuropathy, poems syndrome

## Abstract

POEMS (polyneuropathy, organomegaly, endocrinopathy, monoclonal gammopathy, and skin changes) syndrome remains poorly understood. It is a paraneoplastic syndrome caused by underlying plasma cell dyscrasia, and there is uncertainty about the features required to establish the diagnosis, treatment efficacy, and prognosis. Almost all patients have polyneuropathy or plasma cell disorder, and approximately one-third have Castleman's disease. Peripheral neuropathy often serves as the primary presenting complaint. While extensive discussions exist regarding POEMS syndrome diagnosis and treatment advancements, literature reports of heart failure symptoms associated with POEMS syndrome are scarce. In this article, we present a case of newly diagnosed POEMS syndrome in a 47-year-old man who initially presented with bilateral lower extremities edema and weakness. Although the initial workup was suggestive of heart failure, subsequent evaluation revealed monoclonal gammopathy, sclerotic bone lesions, and Castleman's disease, ultimately leading to the diagnosis of POEMS syndrome.

## Introduction

POEMS (polyneuropathy, organomegaly, endocrinopathy, monoclonal gammopathy, and skin changes) syndrome, also known as osteosclerotic myeloma, Crow-Fukase syndrome, or Takatsuki syndrome is a rare paraneoplastic syndrome associated with plasma cell dyscrasias [1]. The first documented case of what is now known as POEMS syndrome was Scheinker's autopsy case in 1938, and Bardwick et al. created the acronym POEMS in 1980 to represent a syndrome that includes polyneuropathy, organomegaly, endocrinopathy, M protein, and skin changes [2]. However, the acronym does not include certain associated features, such as sclerotic bone lesions, Castleman's disease, papilledema, thrombocytosis, peripheral edema, ascites, effusions, fatigue, clubbing, weight loss, polycythemia, and hyperhidrosis. Not all of these features are necessary for diagnosis.

The pathogenesis of POEMS syndrome is thought to involve the overproduction of inflammatory cytokines, such as TNF-α, IL-1β, IL-6, and vascular endothelial growth factor (VEGF) [3]. It predominantly affects males and typically presents in the fifth or sixth decade of life. Diagnosing POEMS syndrome can be challenging because no single test definitively establishes the diagnosis. Furthermore, because the disease affects multiple systems, its signs and symptoms may not be obvious at the initial presentation, causing diagnostic delays.

POEMS syndrome is sometimes mistaken for other conditions, such as monoclonal gammopathies and chronic inflammatory demyelinating polyneuropathy, both of which can present with peripheral neuropathy [4]. We present the case of a patient who initially presented with heart failure exacerbation and lower extremity weakness, ultimately leading to the diagnosis of POEMS syndrome.

## Case presentation

A 47-year-old man with a medical history of hypertension and chronic kidney disease (stage 1) presented to our emergency room with complaints of progressive bilateral lower extremity weakness, swelling, heaviness, and exertional dyspnea for a year duration. He attributed his weakness to the heaviness caused by the edema. He had not seen a primary care physician in over a year due to the COVID-19 pandemic.

On arrival, his vital signs were as follows: blood pressure, 124/83 mm Hg; heart rate, 78 beats per minute; respiratory rate, 19 breaths per minute; oxygen saturation, 100% on room air and he was afebrile.

Physical examination revealed rales on auscultation of the lung, bilateral lower extremity pitting edema extending to the knees, significant muscle atrophy and decreased reflexes in lower extremities bilaterally and bilateral foot drop. Laboratory findings revealed a B-type natriuretic peptide (BNP) level of 248 pg/ml (reference range: <100 pg/ml), a creatinine level of 1.510 mg/dl (reference range: 0.700-1.300 mg/dl), an albumin level of 3.2 g/dl (reference range: 3.4-5.0 g/dl), an alanine aminotransferase (ALT) level of 12 units/L (reference range: <61 units/L), and an aspartate aminotransferase (AST) level of 4 units/L (reference range: <37 units/L).

**Figure 1:**
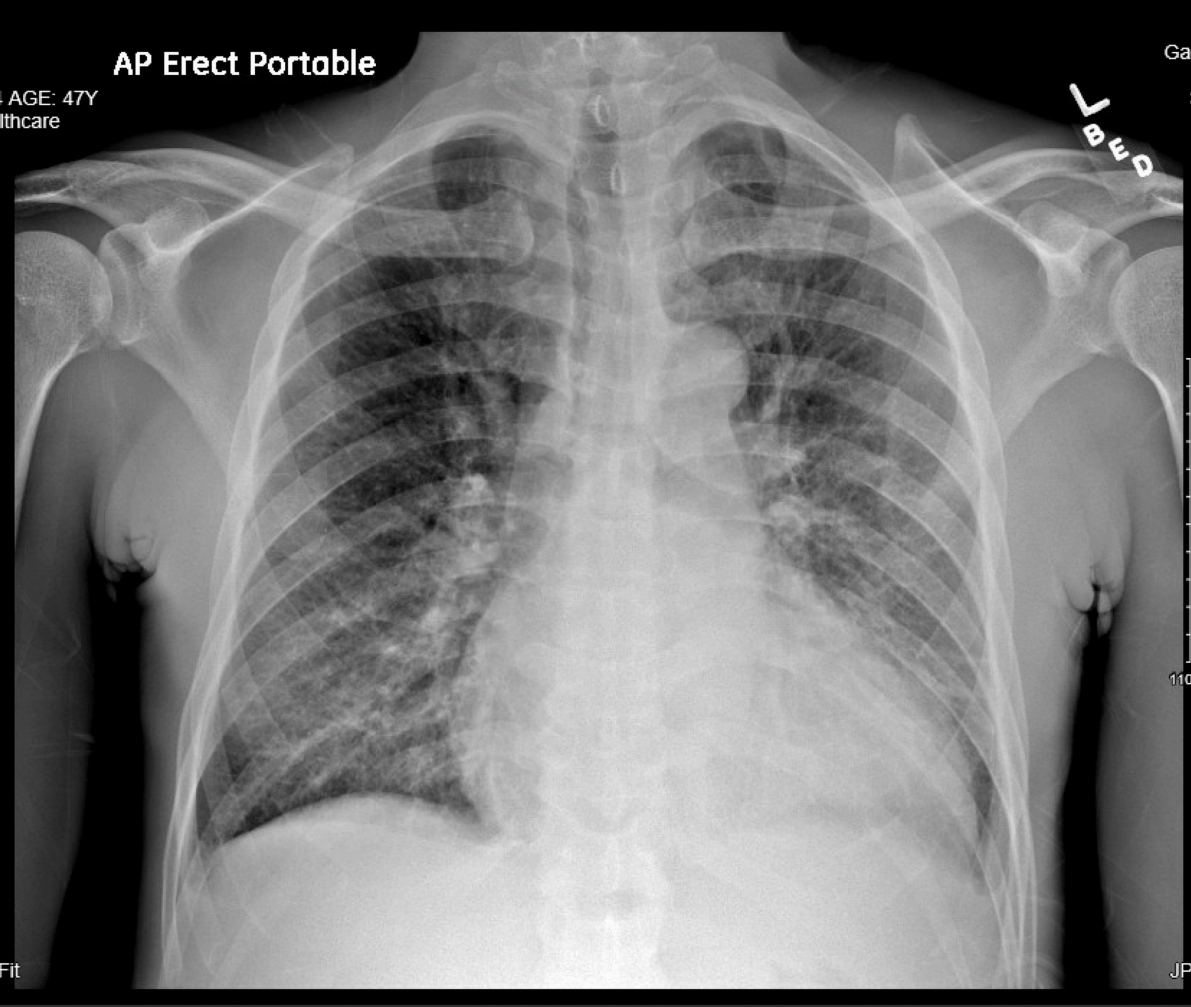
Chest radiograph, in an anteroposterior view. Enlarged cardiac silhouette, accompanied by bilateral increased interstitial markings and cephalization of pulmonary vessels, indicative of pulmonary vascular congestion.

A single anteroposterior chest radiograph was performed that showed cardiomegaly with pulmonary vascular congestion (Figure 1).

**Figure 2:**
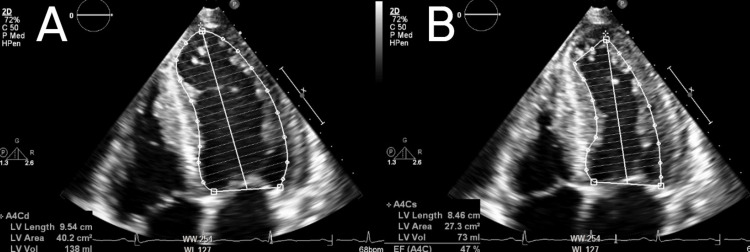
Apical four-chamber view which was used in Simpson's biplane method of disks to estimate mildly reduced left ventricular ejection fraction of 47% on echocardiogram. (A) Image shows left ventricular end-diastolic volume; (B) image shows left ventricular end-systolic volume.

**Figure 3:**
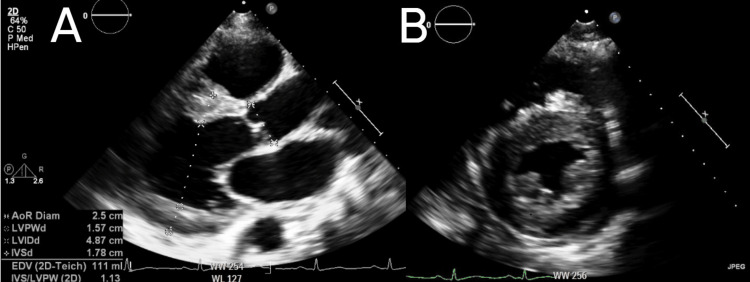
Echocardiogram demonstrating evidence of moderate concentric left ventricular hypertrophy with normal LV cavity size. (A) Parasternal long-axis view on echocardiogram; (B) parasternal short-axis view on echocardiogram. LV: left ventricle.

A transthoracic echocardiogram demonstrated left ventricular hypertrophy (Figures 2A, 2B) with a mildly reduced left ventricular ejection fraction of 47% (Figures 3A, 3B) and normal right ventricular size and function.

As a result, the patient was admitted to the medical floors due to difficulty walking caused by lower extremity edema related to decompensated heart failure. Diuresis was initiated with intravenous frusemide 80 mg daily. However, despite improvements in his respiration and lower extremity edema, his weakness persisted.

An MRI of the brain and spine was performed to rule out central causes of weakness and yielded unremarkable findings. The patient was then discharged to a sub-acute rehabilitation center, with a scheduled follow-up appointment with neurology for further evaluation.

**Figure 4:**
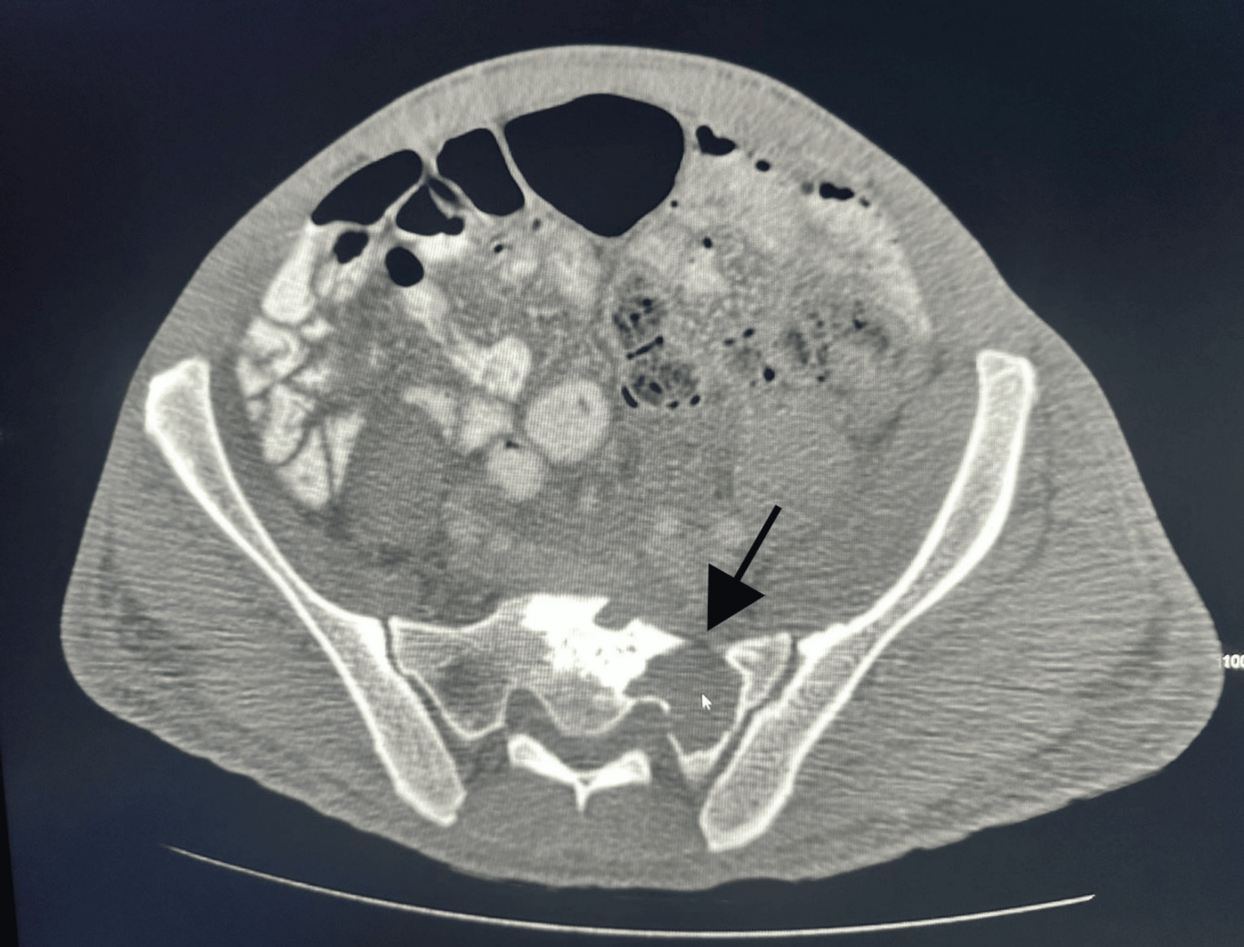
CT of the abdomen and pelvis showing a large lytic lesion in the left sacral bone (black arrow). CT: computed tomography.

**Figure 5:**
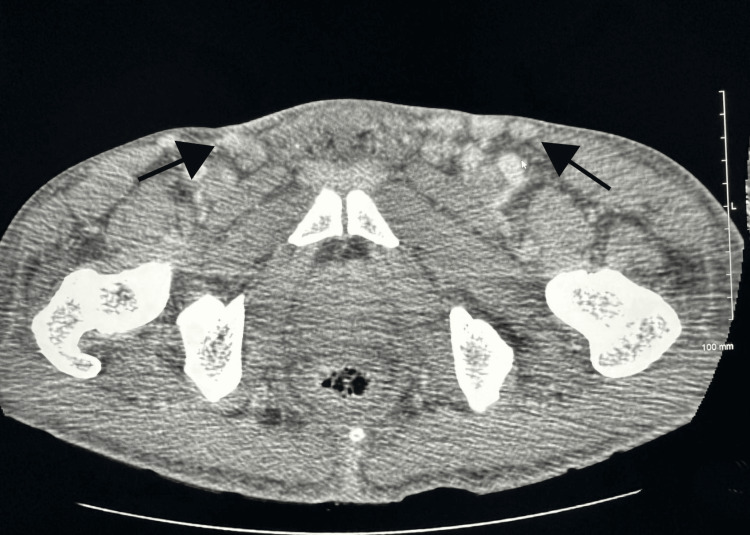
CT of the abdomen and pelvis showing enlarged inguinal lymph nodes (black arrows). CT: computed tomography.

Three months later, and just before his follow-up appointment, the patient returned to the emergency room with new symptoms. He reported increased abdominal girth, discomfort, and an inability to open his left hand fully. We noticed skin hyperpigmentation, persistent bilateral foot drop, and bilateral claw hand deformity, which was worse on the left hand. An abdominal CT scan revealed anasarca and a 5 cm sacral soft tissue mass. In addition, a lytic lesion was found at the lumbosacral spine (Figure 4), as well as bilateral inguinal and pelvic lymphadenopathy (Figure 5).

**Figure 6:**
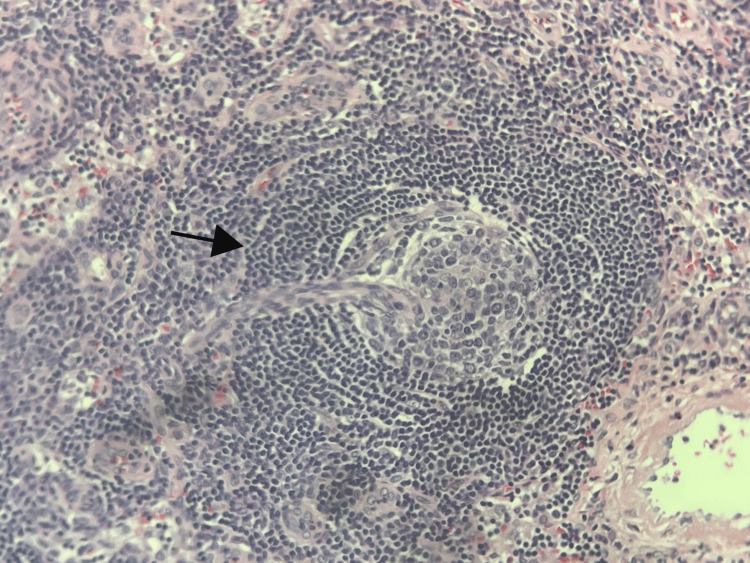
Axillary lymph node biopsy showing mantle zones arranged in concentric rings (black arrow) and penetrating sclerotic blood vessels characteristic of hyaline vascular Castleman's disease.

**Figure 7:**
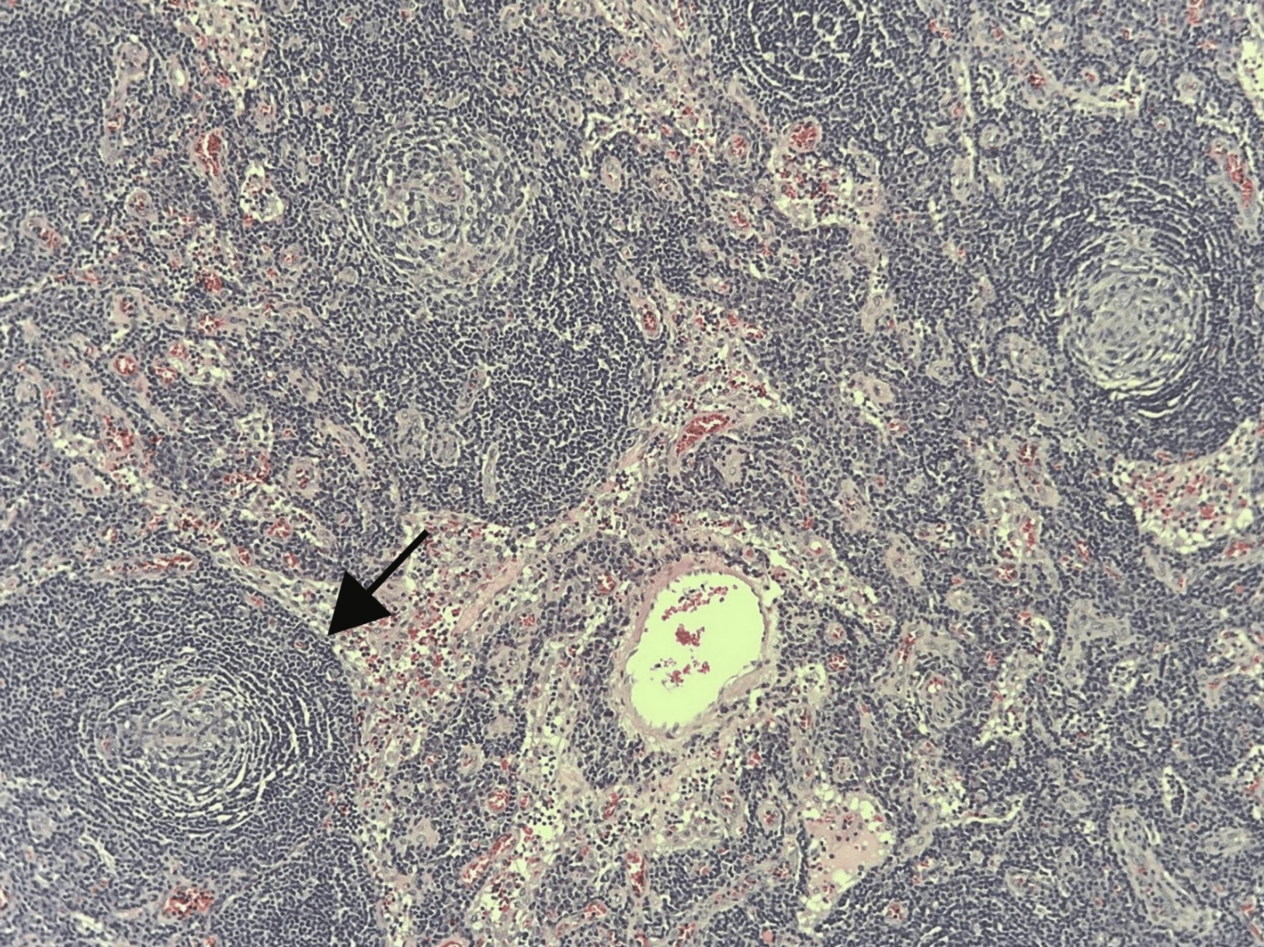
Axillary lymph node biopsy showing germinal centers with concentric onion-like layering of surrounding lymphoid cells (black arrow) seen in Castleman's disease.

The initial differential diagnosis for the patient's weakness included peripheral neuropathy caused by chronic inflammatory demyelinating polyneuropathy, toxic exposure, vitamin deficiency, or monoclonal gammopathy. To further investigate, additional tests were performed to rule out the aforementioned differential diagnoses. These revealed vitamin B12 deficiency with vitamin B12 level of 181 pg/ml (reference range: 193-986 pg/ml), and serum protein electrophoresis (SPEP) showed an elevated spike in the gamma globulin region with a predominance of IgG. The patient underwent a CT-guided biopsy of the sacral mass, which revealed IgG-lambda restricted plasmacytoma. He also had an excisional axillary lymph node biopsy, which revealed histologic characteristics consistent with Castleman's disease, specifically the hyaline vascular variant (Figures 6, 7). Notably, human herpesvirus-8 (HHV-8) immunostaining yielded negative results.

Further testing revealed a remarkably elevated vascular endothelial growth factor receptor (VEGFR) level of 8243. The patient was diagnosed with POEMS syndrome based on a constellation of symptoms and signs including polyneuropathy, monoclonal gammopathy, Castleman's disease, volume overload, skin changes, weight loss, and low vitamin B12 levels.

Initially, the patient received treatment with bortezomib, dexamethasone, and vitamin B12 injections. However, bortezomib had to be discontinued due to severe neuropathy. Subsequently, the patient was switched to daratumumab, lenalidomide, and dexamethasone, resulting in improvements in lower extremity edema, dyspnea, walking ability, and overall functional status. Later, the patient underwent stem cell transplantation, which led to significant improvement. Currently, the patient is receiving monthly maintenance subcutaneous injections of daratumumab.

## Discussion

Diagnosing POEMS syndrome can be extremely challenging, even when a patient presents with a classic set of symptoms. A review of the literature shows that diagnosis of POEMS syndrome is usually delayed by approximately 15 months often it is misdiagnosed with chronic inflammatory demyelinating polyneuropathy and metastatic cancer [5]. Typically, in cases involving peripheral neuropathy, further investigation may lead to the suspicion of POEMS syndrome. However, if neuropathy is not prominent, the diagnosis might be missed unless there is a high level of suspicion.

The diagnostic criteria for POEMS syndrome include two mandatory criteria and three major criteria, one of which must be present. Additionally, there are six minor criteria, one of which must also be met [6]. The mandatory criteria encompass polyneuropathy and monoclonal gammopathy. The major criteria include Castleman's disease, sclerotic bone lesions, and elevated VEGF levels. The minor criteria involve organomegaly, volume overload, endocrinopathy, skin changes, papilledema, and thrombosis. Additional markers, such as low vitamin B12 levels, can provide further support for the diagnosis.

Despite the extensive investigation into various aspects of POEMS syndrome, there remains a significant gap in comprehensive data regarding cardiac involvement. To address this limitation, we have included a table from studies in our literature review in this paper to shed further light on this aspect (Table 1).

**Table 1:** Literature review of POEMS cases presenting with symptoms of heart failure. CIDP: chronic inflammatory demyelinating polyneuropathy; POEMS: polyneuropathy, organomegaly, endocrinopathy, monoclonal gammopathy, and skin changes.

Reference	Age, gender	Presenting symptoms	Initial presumed diagnosis	Cardiac involvement
Pei et al. (2015) [7]	61-year-old, female	Fingers paresthesia and lower limb edema	No initial diagnosis was made	Left ventricular hypertrophy with moderate systolic dysfunction
Witoonpanich et al. (2005) [8]	16-year-old, male	Bilateral lower extremity weakness	CIDP	Left ventricular enlargement with slightly reduced left ventricular systolic function
Wang et al. (2022) [9]	31-year-old, female	Chest pain, dyspnea, and lower extremity edema	No initial diagnosis was made	Left ventricular diastolic dysfunction, pericardial effusion, enlarged left atria
Yokokawa et al. (2013) [10]	67-year-old, male	Dyspnea and lower extremity edema	Heart failure	Cardiomegaly, left ventricular diastolic dysfunction and severe tricuspid regurgitation
Current case	47-year-old, male	bilateral lower extremity weakness and edema and dyspnea	Heart failure	Left ventricular hypertrophy with mildly reduced left ventricular ejection fraction

Our review of the literature shows that cardiac involvement in POEMS syndrome can manifest in diverse ways, including left ventricular hypertrophy, systolic dysfunction, diastolic dysfunction, pericardial effusion, and arrhythmias. It is important to recognize that no single presentation or pattern is specific enough to definitively suspect POEMS syndrome.

In our patient’s case, despite later fulfilling the diagnostic criteria, the initial presentation did not immediately suggest investigating POEMS syndrome. Initially, he presented with volume overload, which, along with his long-standing hypertension and echocardiogram findings, was attributed to congestive heart failure exacerbation. Interestingly, the lower extremity edema also masked his weakness, as he primarily perceived it as heaviness related to the edema and physical deconditioning.

However, as other systemic signs arose and neuropathy became more prominent, particularly with the resolution of the edema and the development of claw hands, further investigations were initiated to explore other potential causes. It is essential to note that volume overload, a symptom commonly seen in heart failure, is also part of the minor criteria in POEMS syndrome.

Vitamin B12 deficiency also poses an additional challenge in diagnosing POEMS syndrome. As its presence can complicate the diagnostic process, vitamin B12 deficiency alone can also cause peripheral neuropathy, further adding to the complexity of the diagnosis.

## Conclusions

While there is a well-defined diagnostic criterion for identifying POEMS syndrome, there is no single symptom or test that would raise the initial suspicion for POEMS syndrome. it is crucial to maintain a vigilant approach to initiate the necessary investigations and meet the diagnostic requirements. When encountering symptoms and signs like peripheral neuropathy and monoclonal gammopathy in the context of heart failure, clinicians should consider the possibility of POEMS syndrome.
